# The sleep and circadian problems of Huntington’s disease: when, why and their importance

**DOI:** 10.1007/s00415-020-10334-3

**Published:** 2020-12-23

**Authors:** Z. Voysey, S. V. Fazal, A. S. Lazar, R. A. Barker

**Affiliations:** 1 Department of Clinical Neurosciences, John Van Geest Centre for Brain Repair, University of Cambridge, Cambridge, UK; 2 Faculty of Medicine and Health Sciences, University of East Anglia, Norwich, UK; 3 Department of Clinical Neurosciences, John Van Geest Centre for Brain Repair, WT-MRC Cambridge Stem Cell Institute, University of Cambridge, Cambridge, UK

**Keywords:** Huntington’s disease, Sleep, Circadian rhythm, Neurodegeneration, Dementia

## Abstract

**Introduction:**

Mounting evidence supports the existence of an important feedforward cycle between sleep and neurodegeneration, wherein neurodegenerative diseases cause sleep and circadian abnormalities, which in turn exacerbate and accelerate neurodegeneration. If so, sleep therapies bear important potential to slow progression in these diseases.

**Findings:**

This cycle is challenging to study, as its bidirectional nature renders cause difficult to disentangle from effect. Likewise, well-controlled intervention studies are often impractical in the setting of established neurodegenerative disease. It is this that makes understanding sleep and circadian abnormalities in Huntington’s disease (HD) important: as a monogenic fully penetrant neurodegenerative condition presenting in midlife, it provides a rare opportunity to study sleep and circadian abnormalities longitudinally, prior to and throughout disease manifestation, and in the absence of confounds rendered by age and comorbidities. It also provides potential to trial sleep therapies at a preclinical or early disease stage. Moreover, its monogenic nature facilitates the development of transgenic animal models through which to run parallel pre-clinical studies. HD, therefore, provides a key model condition through which to gain new insights into the sleep-neurodegeneration interface.

**Conclusions:**

Here, we begin by summarising contemporary knowledge of sleep abnormalities in HD, and consider how well these parallel those of Alzheimer’s and Parkinson’s as more common neurodegenerative conditions. We then discuss what is currently known of the sleep-neurodegeneration cyclical relationship in HD. We conclude by outlining key directions of current and future investigation by which to advance the sleep-neurodegeneration field via studies in HD.

##  Introduction

The relationship between sleep and neurodegeneration is a key focus of contemporary neuroscience due to mounting evidence of an important feedforward cycle between the two.

In one arm of the cycle, neurodegenerative conditions beget sleep abnormalities. As a result of a combination of hypothalamic and brainstem dysfunction, along with a loss of long range connectivity, sleep becomes markedly impaired in 40–90% of patients with neurodegenerative conditions [1–5]. This most commonly manifests as insomnia [4, 6, 7], but frequently also as circadian rhythm dysfunction [8] or other clinical sleep pathologies such as sleep apnoea or rapid eye movement sleep behaviour disorder (RBD) [4]. Such sleep disruption is a crucial determinant of a patient’s quality of life [9], falls risk [10], care giver burden [11] and progression to institutional care [12–15], yet is often not addressed in clinical care.

In the other arm, sleep impairment appears to exacerbate neurodegeneration. Sleep deprivation leads to cognitive deficits in domains including executive function, attention and processing speed, as well as affective disturbances such as impulsivity and emotional lability [16–20]. Sleep abnormalities hence almost certainly contribute to such features common in neurodegenerative conditions. However, increasing evidence contends that abnormal sleep also drives the neurodegenerative pathology directly. For example, sleep deprivation has been found to induce neuroinflammation [21] and aberrant protein homeostasis [22] implicated in neurodegenerative pathophysiology. Likewise, slow wave sleep plays a critical role in memory consolidation through synaptic modulation and the translation of hippocampal information to cortical engrams [23–25]. Sleep loss thus also likely contributes to the synaptic dysfunction and memory impairment of many neurodegenerative diseases. Most significant of all has been the discovery of the glymphatic pathway [26]: a glial-lined perivascular network of channels that allows cerebrospinal fluid to drain interstitial brain tissue of waste solutes. The predominant activity of this pathway in slow wave sleep [27, 28] has revealed the crucial role sleep plays in clearance of neurotoxic proteins. Both beta-amyloid and tau are now known to be cleared by glymphatic activity [29, 30], and sleep deprivation has been consequently been shown to cause marked, rapid increases in these pathogenic protein species [31, 32].

Such findings raise a further question: whether this deleterious feedforward cycle begins with neurodegeneration or abnormal sleep, and thus whether chronic sleep deprivation predisposes to neurodegeneration. In support of the latter, the sleep abnormalities associated with neurodegenerative disease consistently predate symptom onset [7], chronic sleep deprivation has been associated with an almost 50% increase in risk of dementia in large scale epidemiological studies [33, 34], and seminal studies in chronic sleep disruption among airline staff have suggested a causative relationship [35, 36]. This is a question of vital importance to public health, given the endemic societal presence of chronic sleep poverty [37].

Understanding this cycle and treating sleep dysfunction may, therefore, present an important means by which to mitigate the severity and progression, or even onset, of neurodegenerative disease.

Yet, the bidirectional nature of the relationship between sleep and neurodegeneration makes it challenging to study, as cause is difficult to disentangle from effect. Moreover, studies are often weakened by age and comorbidity confounds typical in patients with neurodegenerative conditions, and well-controlled intervention studies are often impractical in the setting of established neurodegenerative disease.

It is this that places Huntington’s disease (HD) as a critical ‘gateway’ condition through which to advance our understanding of the sleep-neurodegeneration interface. HD is a fully penetrant, monogenic, autosomal-dominant inherited neurodegenerative condition, caused by a polyglutamine expansion repeat in exon 1 of the *huntingtin* gene and characterised by a triad of motor, cognitive and psychiatric features typically manifesting between the ages of 35–50. Sleep abnormalities are highly prevalent [38], and, as in other neurodegenerative conditions, precede clinical onset of disease [39]. HD, therefore, provides a crucial opportunity to probe the sleep-neurodegeneration relationship longitudinally, from prior to symptom onset, through the transition phase and into established disease, facilitating a precise interrogation of the strength, timing and directionality of interplay between specific sleep abnormalities and features of neurodegeneration. Moreover, it allows such studies to be undertaken in the absence of age and comorbidity confounds, and offers feasible opportunity to trial therapeutic interventions in prodromal and/or early stage cohorts. Furthermore, its monogenic nature also facilitates the development of transgenic animal models through which to run parallel pre-clinical studies. This is in stark contrast to more common conditions such as Alzheimer’s and Parkinson’s disease, where (1) typically a premorbid phase can only be recognised in hindsight, (2) a definite diagnosis is challenging to resolve with certainty during life, and (3) transgenic animal models cannot recapitulate the predominant, non-Mendelian form of disease.

The study of sleep disturbance in HD, therefore, bears vital potential to inform the wider sleep-neurodegeneration field. Here, we begin by summarising contemporary knowledge of sleep abnormalities in HD, and consider how well these parallel those of Alzheimer’s and Parkinson’s as more common neurodegenerative conditions. We then discuss what is currently known of the sleep-neurodegeneration cyclical relationship in HD, and the evidence as to how HD causes sleep abnormalities, and whether sleep abnormalities drive HD pathophysiology. We conclude by outlining key directions of current and future investigation by which to advance the sleep-neurodegeneration field via studies in HD.

## Sleep and circadian abnormalities in Huntington’s disease: evidence to date

### Clinical sleep pathology

Epidemiological analysis has suggested that clinical sleep pathology is present in as many as 88% of patients with manifest HD [40]. However, such observations are based on subjective patient reports, and studies comparing these to formal sleep study assessments have revealed prominent discrepancy, both with respect to over- and under-estimation [41, 42]. It is thus judicious to limit any review only to those studies incorporating objective sleep assessments when considering clinical sleep pathologies characterising HD. Of these, the consensus emergent from the literature is of two prominent pathologies: insomnia [43, 44] and abnormal motor activity during the sleep period [43, 45–48]. There is controversy as to what this motor activity constitutes [49, 50]: two studies have identified evidence of frequent periodic limb movements during sleep [43, 44], but in addition, several studies have identified complex, high amplitude and non-stereotyped movements that occur subsequent to arousals to wakefulness [46–48]. These have been interpreted as chorea [47, 48], repetitive ballistic movements [47], or voluntary repositioning movements rendered atypical by underlying HD-related hypotonia and incoordination [46]. RBD does not appear to be prevalent [43, 44]; nor does sleep disordered breathing [43, 51, 52] with the exception perhaps of late stage disease [53]. With respect to excessive daytime somnolence, two studies have identified shortened latencies to sleep on objective daytime nap tests [39, 44] but the majority of studies have found this to be clinically asymptomatic [43, 44, 54].

In this respect, HD fails to closely recapitulate the sleep abnormalities of other more common neurodegenerative conditions. For example, while insomnia is a common denominator across the spectrum of neurodegeneration, Alzheimer’s disease is typified by high rates of sleep disordered breathing [55], while Parkinson’s disease is characterised by prominent RBD and excessive daytime somnolence [56]. Nonetheless, such discrepancies are arguably themselves of value, informing how differing patterns and mechanisms of neurodegeneration give rise to divergent sleep pathologies.

### Sleep architecture abnormalities

Sleep is divided into stages by encephalography (EEG), muscle tone and eye movement hallmarks identifiable during polysomnography (sleep studies; PSG). Healthy adult sleep is highly structured, characterised by a transition through progressively deeper non-rapid eye movement sleep (NREM) stages known as N1, N2 and N3/slow wave sleep, followed by rapid eye movement (REM) sleep. This cycle repeats four to five times per night. Aberrations of this structure, referred to as sleep architecture, are highly prevalent in neurodegenerative disease, even in individuals without clinical sleep pathology [42].

There have been numerous clinical PSG studies in HD [39, 42–44, 46, 57], alongside a number in animal models [58, 59]. There is marked variability among these studies as to the sleep architecture abnormalities that are reported. The reasons for this are likely manifold: a lack of genetic diagnosis in early studies, heterogeneity of clinical stage, medication confounds, use of single night PSG which is vulnerable to habituation artefact, and discrepancy in the PSG features being studied.

However, a recent meta-analysis [60] of these studies has helped to address this. This identified the consistent sleep architecture abnormalities of HD as being those of reduced sleep efficiency, increased time awake after sleep onset, delayed REM sleep onset, and an increased proportion of time spent in the lightest stage of sleep (N1) versus a reduction of time spent in slow wave sleep and REM sleep. Of note, this shares striking similarities with the abnormalities found in Alzheimer’s and Parkinson’s disease [7], supporting the possible validity of HD as a model for more common conditions with respect to sleep architecture changes.

Cross-sectional studies of premanifest and early stage HD patients have revealed such sleep architecture abnormalities to be present many years prior to manifest disease, and to worsen with approaching manifest disease onset [39, 42, 44]. Among the subsequent manifest HD population, sleep abnormalities appear to continue to progress in parallel with increasing disease severity [44, 57, 60]. Again this mirrors findings in Alzheimer’s and Parkinson’s [61, 62].

Some studies have also assessed for the presence of sleep fragmentation, rather than simply the total time spent in each sleep stage [39]. This has revealed that fragmentation appears to be an important component of HD sleep architectural disturbance, including in the premanifest phase. Once again this parallels findings in Alzheimer’s and Parkinson’s, where sleep fragmentation in particular has been identified as a feature of both premonitory and established disease [62–66].

Transgenic HD mouse models [58, 59] have corroborated these findings in a number of notable ways, demonstrating increased sleep fragmentation and a loss of slow wave sleep, and progression of sleep abnormalities with disease severity. However, in contrast to human studies, they demonstrate an increase in REM phase. The latter likely reflects imperfect congruity between human and rodent sleep, and between murine HD models (with much longer polyglutamine repeat length and the use of differing promoters) versus human disease.

A handful of studies have employed quantitative EEG: analysis of the relative frequency composition of PSG EEG recordings. Transgenic HD mouse models have demonstrated an increase in gamma frequency content in NREM sleep, and a shift of REM peak frequency from 7 to 6 Hz [58]. Echoing this, human studies have indicated an increase in gamma content of NREM sleep, and a loss of theta range activity in REM and NREM sleep [39]. Similarly, a recent study employing a technique to localise quantitative EEG changes has also indicated theta component loss: in motor/premotor areas in NREM, and in limbic regions in REM [41]. Evidence is as yet too sparse to place weight on these findings, but it is of note that theta range activity in sleep has been implicated in neural restoration [67], and a gain in gamma frequencies has been recognised in both schizophrenia and drug-induced psychosis, including in sleep [68].

There is thus relatively comprehensive evidence for sleep architecture changes in HD. Yet, the most informative study has yet to be performed: that of a longitudinal study of these abnormalities in an HD patient cohort, initiated in the premanifest phase and tracked against clinical features.

### Circadian abnormalities

Circadian abnormalities are also prevalent in HD, with available evidence favouring a loss of circadian amplitude over a defined phase shift. For example, transgenic animal models of HD have demonstrated progressive dampening of circadian rhythmicity, as indicated by diminished diurnal patterns of activity, heart rate variability and body temperature [58, 69, 70]. Clinical studies have supported this, for example demonstrating loss of circadian blood pressure variability [71] and disrupted rest-activity patterns without overt phase shift [59]. Such blunting of circadian rhythm is shared by Alzheimer’s and Parkinson’s disease, although phase shift appears to also be present in Alzheimer’s [8].

Three cross-sectional studies have examined circadian melatonin release in HD patients. These have failed to corroborate one another, reporting mixed results regarding phase shift, reduction or increased temporal spread in melatonin secretion [54, 72, 73]. This lack of consensus owes as much to the paucity of studies as it does to the low number and heterogeneity of participants within them: a large-scale and longitudinal study of melatonin in an HD cohort is, therefore, needed.

### How Huntington’s disease causes sleep abnormalities

HD neuropathology is known to affect the hypothalamus [74, 75]. Since this is the seat of both the body’s master circadian clock, the suprachiasmatic nucleus, and orexin neurons responsible for gating sleep/wake states, this is postulated as a key mechanism by which HD brings about sleep and circadian dysfunction. In support of this, abnormalities in orexinergic function have been observed [76, 77] and altered neuronal firing rates have been identified in the suprachiasmatic nucleus in HD animal models [78]. Nonetheless, the applicability of such findings to human disease is questionable. For example, there is poor overlap of hypothalamic abnormalities in HD pre-clinical and clinical studies [79], and clinical imaging studies of hypothalamic changes have shown little correlation with sleep/circadian dysfunction [80].

HD neuropathology is also proposed to bring about sleep dysfunction via influence in other brain regions. For example, HD is recognised to lead to atrophy in brainstem structures such as the locus coeruleus that gate transition between sleep stages [81, 82]. Likewise, healthy slow wave sleep requires both local cortical and brain-wide synchrony of neuronal oscillatory activity. It may, therefore, also be that the widespread cortical pathology of HD, which affects connectivity even from presymptomatic stages [83], accounts for deficits in slow wave sleep. Moreover, recent evidence has identified a possible role of the striatum in governing arousability during sleep, and the generation of periodic limb movements [43, 84, 85]. As the striatum is a major early and enduring site of HD pathology, this raises a further possible link between HD pathology and sleep disruption. Direct evidence to support such hypotheses is, however, lacking.

Preclinical models have sought to explain circadian disruption in HD through the effect of HD pathology on clock genes. Clock genes are those that interact via intricate feedback loops generating cycles of gene expression with a period of approximately twenty-fours, thereby providing cellular circadian rhythm. These are expressed in the suprachiasmatic nucleus to provide master, coordinating circadian periodicity, but also function in most nucleated cells to provide cell-autonomous circadian rhythm that is then ‘tuned’ by the master clock and other peripheral cues [86]. Studies of clock genes in transgenic HD mice have almost invariably found evidence of aberrant clock gene expression in vivo, in sites ranging from the suprachiasmatic nucleus to the neocortex to liver [59, 69, 87]. However, significantly, these aberrations resolve upon ex vivo study [87, 88]. This suggests that circadian dysfunction in HD is driven by system-wide influences on clock gene expression, such as altered melatonin, cortisol or circadian behaviour rhythms, rather than the direct influence of mutant huntingtin protein on intrinsic clock gene function at a cellular level. In support of this, a transgenic sheep model of HD found that circadian behaviour abnormalities dissipated when animals were housed with controls providing normal circadian cueing [89], suggesting that circadian dysfunction is driven more by altered extrinsic zeitgebers than by intrinsic clock gene expression alterations. Nonetheless, such findings are again hypothetical, as there have so far not been any studies of clock genes in HD patients.

### Evidence linking Huntington’s sleep abnormalities to disease exacerbation

Most notable of all is the evidence that sleep abnormalities exacerbate HD pathology.

A number of pre-clinical intervention studies have emerged over recent years suggesting that treating sleep abnormalities may improve clinical outcomes in HD. The earliest of these, employing alprazolam in a transgenic HD murine model, indicated that pharmacological imposition of sleep could improve cognitive outcome, independent of anxiolytic effect [88]. A further study by the same group demonstrated that behavioural circadian intervention, mediated by housing animals with intact circadian behaviours, had the same effect [90]. A more recent study of circadian re-entrainment, delivered via blue light therapy in transgenic HD mice, also led to improvements in locomotor activity rhythms and motor performance [91]. However, sleep timing or quantity did not alter, and therefore, it is questionable whether the benefit was secondary to the alerting influence of light therapy rather than its impact upon circadian rhythm or sleep quality. Nonetheless, a further study of light-mediated circadian entrainment in transgenic HD mice has demonstrated improved survival rates [92], and another employing timed feeding as a circadian cue has also reported improved motor outcomes [93]. The most recent pre-clinical study of sleep therapy in HD, using an orexin antagonist, has also demonstrated benefit to cognitive outcome [94].

Such studies are of great significance, as they add weight to the notion that sleep abnormalities exacerbate neurodegeneration, and suggest that sleep therapies may, therefore, present a viable means by which to slow disease progression. Given that we currently have no proven disease-modifying treatments for HD, this is of salient importance.

Nevertheless, none of these studies has yet evaluated whether the interventions modified HD outcomes through changes at a pathophysiological level, or whether these were mediated solely through symptomatic gains. A definitive study of whether sleep deprivation promotes accumulation or spread of mutant huntingtin protein, for example, is lacking.

Moreover, to date, there has been no retrospective and only one prospective translational sleep therapy intervention study in HD. This comprised a 9 month pilot study of a multidisciplinary intervention, including exercise, cognitive training, sleep hygiene measures, nutritional guidance and socialisation, in a cohort of sixteen premanifest HD patients [95]. This intervention was well appointed, given that such multi-component therapies carry some of the greatest evidence for efficacy and safety in treating sleep in neurodegenerative conditions [96–99]. Gains in REM sleep were demonstrated, while improvements in slow wave sleep, HD-related structural brain changes and cognitive outcomes were not observed. While disappointing, it should be noted that the study was limited in a number of ways. Besides its small sample size and lack of control group, pre and post intervention PSG was of one-night duration and, therefore, liable to habituation artefact, and cognitive improvement was assessed via only one cognitive test: the Hopkins Verbal Learning Test of memory consolidation. There is, therefore, a prescient need for larger, better controlled and more detailed clinical studies of sleep therapy intervention in HD. These would be enhanced further by the inclusion of biomarkers of disease activity as endpoints. There is growing contemporary evidence for the validity of serum neurofilament light chain, and cerebrospinal tau and mutant huntingtin, in this regard [100, 101], making this a natural line of investigation yet to be pursued.

## Conclusions and future directions

Though HD differs from more common conditions like Alzheimer’s and Parkinson’s with respect to prevalent clinical sleep pathology, there are important parallels evident in relation to the timing and nature of sleep architecture and circadian disturbances. This suggests that HD constitutes a valid model condition, while offering important opportunities not afforded by other neurodegenerative conditions. As such, Huntington’s disease is well-placed to provide key new insights into the relationship between sleep and neurodegeneration.

Great advances have been made over the last few years to exploit this potential, in particular in the form of pre-clinical sleep intervention studies examining the link between sleep abnormalities and disease exacerbation. The fact that these have demonstrated improved clinical outcomes in response to treating sleep dysfunction raises important hopes both for HD and the wider neurodegeneration field.

However, a number of salient gaps in the literature remain:

Firstly, there has yet to be a longitudinal clinical study of sleep abnormalities versus clinical outcomes in Huntington’s disease, initiated from the premanifest phase. This is a prominent omission, since this is the key opportunity feasible in HD that is not possible in other neurodegenerative conditions. Such a study would enable delineation of the strength and timing of associations between specific sleep abnormalities and features of neurodegeneration, helping to unravel the ‘chicken and egg’ of the sleep-neurodegeneration relationship. This is a study we are currently completing.

Secondly, there has yet to be a study of clock gene expression in HD patients. The most valuable study would be one designed to differentiate aberrant expression due to altered cell-extrinsic cues from that caused by intrinsic clock dysfunction. This is needed to translate the findings of pre-clinical studies, and to resolve the source of circadian dysfunction to in turn guide therapeutic strategies. Akin to this, more detailed studies of the mechanisms by which HD effects sleep architecture abnormalities would also help guide therapy trials.

Thirdly, studies evaluating the effect of sleep disturbance on HD disease activity, rather than solely clinical features, are currently lacking. This could be achieved, for example, by subjecting transgenic HD mice to sleep deprivation or sleep therapy, and assessing the effect on central nervous system mutant huntingtin accumulation. This is vital to understanding whether sleep abnormalities exacerbate HD disease burden on a pathophysiological or a solely symptomatic level.

Finally, and most importantly, there is a pressing need for robust sleep intervention studies in HD cohorts, to assess whether the promising findings of pre-clinical studies translate to patients. Current evidence does not favour the use of traditional sedating agents as sleep interventions in neurodegeneration: studies of antidepressants, antihistamines and antipsychotics in patients with mixed dementia, Alzheimer’s and Parkinson’s, for example, have suggested that deleterious effects outweigh benefit when used as sleep agents [102–104]. Likewise, there is presently a lack of cogent evidence suggesting significant benefit from modafinil [105, 106] or melatonin [107, 108]. However, in addition to the described behavioural interventions [109], sodium oxybate [110, 111], orexin antagonists [112, 113] and devices facilitating acoustic augmentation of slow wave sleep [114] are all demonstrating potential to improve both sleep and cognitive outcomes in neurodegenerative cohorts, but have yet to be trialled in an HD population. Again, the most valuable studies employing such interventions would be those incorporating biomarkers of disease activity as endpoints, to interrogate effects on clinical features versus pathophysiology.

We believe that such studies represent a research priority, both for Huntington’s disease and for informing the wider sleep-neurodegeneration field.
